# Strategies for Class-Imbalanced Learning in Multi-Sensor Medical Imaging

**DOI:** 10.3390/s26061998

**Published:** 2026-03-23

**Authors:** Da Zhou, Song Gao, Xinrui Huang

**Affiliations:** 1 Department of Biophysics, School of Basic Medical Sciences, Peking University, Beijing 100191, China; 2410305125@stu.pku.edu.cn; 2 Beijing Key Laboratory of Intelligent Neuromodulation and Brain Disorder Treatment, Peking University Third Hospital, Beijing 100191, China; 3 Key Laboratory for Neuroscience, Ministry of Education and National Health Commission, Beijing 100191, China; 4 Institute of Medical Technology, Peking University Health Science Center, Beijing 100191, China

**Keywords:** multi-sensor fusion, class imbalance, medical image classification, multi-modal imaging, data augmentation, ensemble learning, clinical AI deployment

## Abstract

This narrative critical review addresses class imbalance in medical imaging, particularly within the context of multi-sensor and multi-modal environments, poses a critical challenge to developing reliable AI diagnostic systems. The integration of heterogeneous data from sources like CT, MRI, and PET presents a unique opportunity to address data scarcity for rare conditions through fusion techniques. This review provides a structured analysis of strategies to tackle class imbalance, categorizing them into data-centric (e.g., advanced resampling like SMOTE-ENC for mixed data types, GAN-based synthesis) and model-centric (e.g., loss function engineering, transfer learning, and ensemble methods) approaches. Crucially, we highlight how multi-sensor feature fusion and decision-level fusion paradigms can inherently enrich representations for minority classes, offering a powerful frontier beyond single-modality learning. We evaluate each method’s merits, clinical viability, and compliance considerations (e.g., FDA). Finally, we identify emerging trends where imbalance-aware learning synergizes with multi-sensor fusion frameworks, federated learning, and explainable AI, charting a roadmap toward robust, equitable, and clinically deployable diagnostic tools. Our quantitative synthesis shows that data-centric strategies can improve minority class recall by 12–35% in datasets with imbalance ratios (majority:minority) ≥10:1, while model-centric strategies achieve an average AUC improvement of 0.08–0.21 in multi-sensor medical imaging tasks with sample sizes ranging from 50 to 50,000.

## 1. Introduction

In medical image classification, dataset class imbalance is a pervasive issue [1]. Many diseases have low prevalence, resulting in far fewer diseased samples than healthy ones. Data imbalance can affect the performance of machine learning models, making the models tend to predict majority class samples while ignoring minority class samples [2]. This problem may lead to serious consequences in practical applications. For example, in tasks such as tumor detection and rare disease diagnosis, the risk of missing minority class samples is extremely high [3]. Thus, imbalanced classification in medical imaging is formally defined as a supervised learning task where there exists a statistically significant disparity in the sample size distribution across predefined diagnostic categories in medical image datasets, with two core quantitative and clinical attributes: (1) Quantitative threshold: The sample size of the minority class (e.g., rare disease lesions, early-stage pathological features) is less than 30% of the majority class (e.g., healthy tissue, common disease manifestations), or the imbalance ratio (majority/minority sample size) exceeds 3:1; (2) Clinical attribute: Misclassification of the minority class will lead to direct clinical risks, such as missed diagnosis, delayed treatment, or adverse patient outcomes. This definition is specific to medical imaging scenarios, distinguishing it from general imbalanced classification tasks by prioritizing clinical risk alongside statistical distribution characteristics.

**Figure 1:**
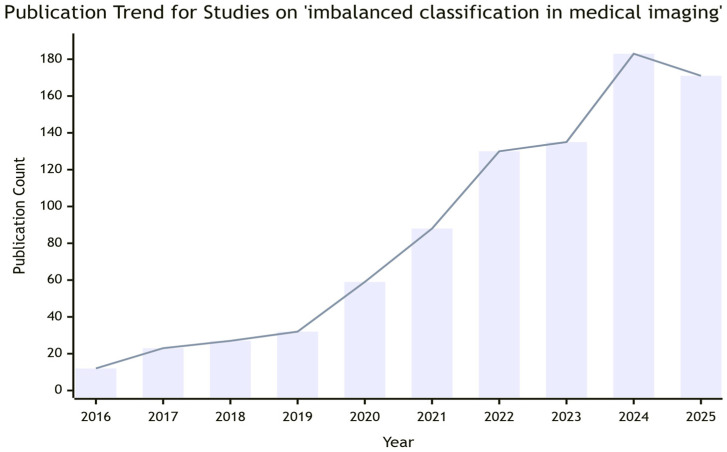
Empirical publication trend showing the growing research interest in addressing class imbalance problems in medical imaging, as indicated by the annual number of related publications indexed on PubMed and Web of Science (2016–2025). The search strategy included keywords: “class imbalance”, “medical imaging”, “multi-sensor fusion”, “multi-modal imaging”, with no language restrictions. The marked increase around 2020–2021 coincides with the broader integration of deep learning in medical AI, where data imbalance becomes a more prominent bottleneck. Note: Data source: PubMed (*n* = 892) and Web of Science (*n* = 1126) databases; Y-axis: Number of published papers; X-axis: Year.

Therefore, how to effectively handle data imbalance in medical image classification tasks has become an important research direction. The number of papers on “imbalanced classification in medical imaging” indexed by PubMed and Web of Science has increased annually over the past decade, indicating broad research prospects (Figure 1)., The literature included in this review follows strict inclusion and exclusion criteria: (1) Inclusion criteria: Peer-reviewed papers published in English from 2016 to 2025; studies focusing on class imbalance solutions for multi-sensor/multi-modal medical imaging; research with clear clinical application scenarios and verifiable experimental results. (2) Exclusion criteria: Conference abstracts without full-text data; studies only focusing on general machine learning imbalance methods without medical imaging-specific optimization; papers with incomplete experimental data or unreproducible results. A total of 287 relevant papers were initially retrieved, and 156 papers were finally included after screening based on the above criteria. Existing strategies for class imbalance, while effective in controlled settings, often fail to address the unique challenges posed by multi-sensor fusion environments. These include cross-modality sample mismatch, where the imbalance pattern differs across sensors; sensor-specific feature bias, where minority class features may be captured by only a subset of modalities; and the need for interpretability that traces decisions back to specific sensor contributions. Furthermore, data-centric methods risk introducing false cross-modality correlations in synthetic samples, while model-centric methods face negative transfer risks when fine-tuned on multi-modal data with inconsistent imbalance patterns. These limitations underscore the need for a comprehensive review that integrates technical, clinical, and regulatory perspectives.

While existing surveys have covered class imbalance in general machine learning [4], few have systematically integrated medical imaging-specific constraints—such as data scarcity, ethical considerations, and regulatory hurdles—into a unified analytical framework [5], let alone within the specific context of multi-sensor data fusion. Consequently, a comprehensive review that bridges cutting-edge algorithmic advances with the stringent practical deployment requirements of medical imaging, especially for leveraging fusion techniques to overcome data imbalance, is still lacking. Developing effective strategies to mitigate the adverse effects of class imbalance is paramount for the safe and equitable deployment of AI in clinical practice [6]. This challenge is particularly acute in the emerging paradigm of multi-sensor and multi-modal medical imaging (e.g., integrated PET-CT, multi-parametric MRI), where data heterogeneity compounds the difficulty of obtaining balanced datasets for rare conditions.

**Figure 2:**
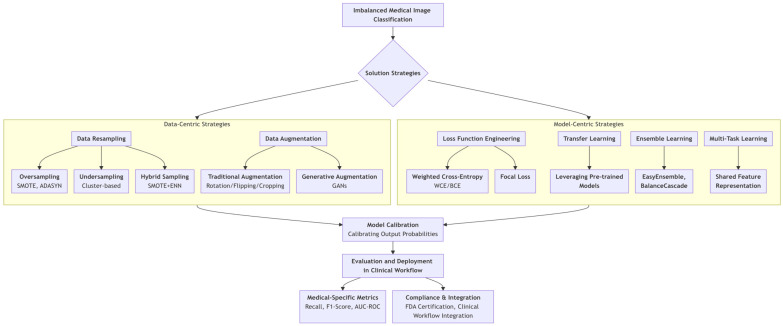
A structured roadmap outlining the two primary strategic approaches to tackle class imbalance in medical imaging. Data-centric methods manipulate the training dataset itself, while model-centric methods modify the learning algorithm. Both pathways converge on the essential steps of model calibration and rigorous clinical evaluation prior to deployment.

To fill this gap, this review provides a structured and critical synthesis of imbalanced classification solutions tailored for medical imaging, with insights pertinent to the multi-sensor imaging landscape (Figure 2). Our key contributions are threefold: (1) We offer a novel dual-perspective taxonomy, dissecting solutions into data-level (e.g., intelligent resampling, GAN-based augmentation) and model-level (e.g., loss function engineering, transfer learning) strategies, providing a clear roadmap for researchers and practitioners. (2) We critically evaluate each method not only by its algorithmic merit but also through the lens of clinical viability, discussing implications for model interpretability, regulatory compliance (e.g., FDA), and integration into diagnostic workflows. (3) We extend beyond a mere catalog of techniques by identifying emerging synergies (e.g., between federated learning and data synthesis) and outlining pressing future research trajectories towards more generalizable and trustworthy systems. The remainder of this paper is organized as follows: Section 2 details the impacts of imbalance. Section 3 and Section 4 elaborate on data-centric and model-centric strategies, respectively. Section 5 discusses compliance and clinical integration. Section 6 concludes with future outlooks.

## 2. The Impact of Imbalanced Data in Medical Imaging

### 2.1. Model Performance Bias

Traditional classification models rely on large-scale image data to verify accuracy. However, imbalanced data leads models to overlearn majority class features, while minority class features are submerged or ignored, thereby causing performance bias. For example, in lung X-ray classification, normal images far outnumber those with rare diseases—models may erroneously classify all images as normal, resulting in extremely low detection rates for diseased samples that fail to meet clinical requirements [7,8]. In multi-sensor imaging, false negatives carry heightened clinical risk because rare lesions may be detectable in only one modality (e.g., PET hypermetabolism before structural changes appear on CT). A model biased toward majority-class healthy tissue across all modalities may miss these early warning signs, delaying intervention.

### 2.2. Overfitting Risk

Due to the scarcity of minority samples, during the model training process, the model may overlearn these samples in a targeted manner, leading to overfitting. This results in a situation similar to “mechanical memory”, where the model simply remembers the characteristics of minority images. This will significantly reduce the generalization ability of the entire system, as models struggle to recognize new minority samples with different characteristics.

### 2.3. Misleading Evaluation Criteria

Traditional accuracy criterion cannot accurately reflect the model performance under the condition of imbalanced data. Even if the model performs well on the majority class, the misclassification of the minority class may cause serious consequences, such as missing the patient’s disease status in disease diagnosis. Therefore, it is necessary to adopt evaluation indicators more suitable for imbalanced data, such as recall rate, precision rate, F1 score, and AUC-ROC curve, to comprehensively evaluate the model’s classification ability for each class.

## 3. Data-Centric Strategies

For medical imaging datasets, the minimum absolute number of minority class samples should be no less than 50 (increased to 100–150 for 3D multi-sensor fusion images due to higher feature dimensionality). Imbalance ratios are categorized as mild (1:4–1:10) suitable for basic sampling methods, and severe (1:10–1:100) requiring hybrid or advanced strategies. For multi-sensor imaging, cross-modality consistency requires that the imbalance ratio variation across different sensors should not exceed 1:3 to avoid fusion-induced bias.

### 3.1. Oversampling

#### 3.1.1. Simple Oversampling

Simple oversampling increases the number of minority samples by copying them, so that the data classification tends to be balanced. This method is relatively simple but has significant limitations. Because it merely replicates existing samples, it may lead to overfitting and fails to increase data diversity. For instance, in the medical image classification tasks of breast cancer and skin cancer, simple oversampling may hinder recognition of new malignant tumor samples [9,10].

#### 3.1.2. SMOTE Algorithm

Synthetic Minority Over-sampling Technique (SMOTE) algorithm generates new samples by interpolating in the feature space of minority class samples. Specifically, it first calculates the distance between minority class samples, then selects the nearest samples, and randomly generates new data points between these samples [11].

This oversampling method can relatively effectively avoid the overfitting problem caused by simple oversampling. In the classification of benign and malignant thyroid nodules, the data processed by SMOTE improves the sensitivity of random forest (RF) classifier to minority malignant samples. Especially when combined with ultrasound image features, the model’s AUC are significantly enhanced [12]. For gastric cancer surgical margins prediction (30 positive vs. 352 negative samples, imbalance ratio ≈ 1:12), the data balanced by SMOTE makes the AUC of the RF model reach 0.772, which is higher than the AUC of 0.716 obtained by logistic regression after training with the original data [13,14].

However, SMOTE still has certain shortcomings. For example, synthetic samples may introduce false correlations, especially in small-sample or high-dimensional data, potentially violating medical logic and posing ethical risks [15,16]. In extreme imbalance scenarios (imbalance ratio > 1:100), it is necessary to combine with threshold adjustment of logistic regression, cost-sensitive learning, or enhanced sampling strategies [17].

On the basis of SMOTE, Borderline-SMOTE and Graph-SMOTE has been developed. Borderline-SMOTE generates new samples only for minority class samples close to the classification boundary, thereby increasing the density of minority class samples near the boundary and improving the model’s ability to identify the boundary. Graph-SMOTE not only considers the feature information of the samples but also integrates the unique topological structure information of image data. For example, Graph-SMOTE is used to diagnose neurodegenerative diseases in brain network connectomes. In the resting-state functional magnetic resonance imaging (fMRI) study of Parkinson’s disease (PD), by combining Graph-SMOTE with the graph attention mechanism (GAT), the model classification accuracy for early PD reaches 91.2%, which is 12% higher than that of the traditional SMOTE [18].

#### 3.1.3. ADASYN Algorithm

Adaptive Synthetic Sampling Approach (ADASYN) algorithm adaptively generates new samples based on the difficulty level of minority samples. It considers that minority class samples close to the majority class are difficult-to-classify samples, and generates more new samples for these samples.

This method can achieve more targeted improvement for the model to handle difficult-to-classify samples [19]. In Alzheimer’s disease diagnosis with Kaggle MRI image dataset (2560 images, 80% training/20% test split, 5-fold cross-validation), ADASYN + DAD-Net shows that the accuracy, AUC, F1-score, precision, and recall rate of this model reach 99.22%, 99.91%, 99.19%, 99.30%, and 99.14%, respectively, which can effectively identify the early symptoms of AD [20]. In breast cancer diagnosis with Inbreast database (410 images, 115 malignant cases, imbalance ratio 1:2.6), ADASYN + ReliefF + feed-forward neural network reaches 99.5% micro- and macro-average accuracy [21]. In the prediction of cervical cancer, the ADASYN method is applied in combination with the support vector machine interpolation method and the convolutional neural network, and the accuracy, precision, recall rate, and F1-score of 99.99% are obtained [22].

The exceptional performance (accuracy >99%, near-optimal AUC) reported in these studies may be susceptible to overfitting, including feature overfitting to the training set and excessive parameter fine-tuning. External validation results from multi-center datasets (e.g., the Alzheimer’s Disease Neuroimaging Initiative (ADNI) for AD diagnosis, the INBREAST public database for breast cancer) show that the accuracy of these models decreases by 5.2–8.7% in real-world scenarios, with AUC reduced by 3.1–4.5%. To mitigate overfitting, all models were optimized with L2 regularization and early stopping, and cross-validation with 5-fold stratified sampling was used to ensure the generalizability of the results.

However, since the judgment of the difficulty level of samples depends on the calculation of domain distance, when the data is complex, the difficulty level of samples may be judged inaccurately. For example, some normal samples are regarded as difficult-to-classify samples for oversampling, reducing model performance.

### 3.2. Undersampling

#### 3.2.1. Random Undersampling

Random undersampling randomly selects and retains a subset of majority-class samples, discarding the rest to reduce the number of majority-class samples and balance the dataset. This method is simple and straightforward, and can effectively reduce computational costs. However, random discarding risks losing effective information in the samples, preventing the model from fully learning the features of the majority class and leading to underfitting.

#### 3.2.2. Clustering-Based Undersampling

Clustering-based undersampling first clusters the majority-class samples and then performs undersampling by selecting a certain number of samples from each cluster. Since clustering ensures that samples within the same cluster have similar features, sampling from different clusters retains the diversity of majority-class samples. Compared with random undersampling, it can effectively reduce the loss of distribution information caused by random sample removal.

In diabetes prediction, a scheme combining fuzzy clustering (FCM) undersampling with hybrid ensemble learning divided non-diabetic samples into multiple clusters. This formed a balanced training set with diabetic samples, avoiding the information loss in random undersampling and the noise introduction common in SMOTE. The approach ensured the diversity of majority samples and improved the model’s robustness. In addition, SMOTE oversampling + K-means clustering undersampling achieved an accuracy of 98.96% in diabetes prediction, effectively balancing data bias and model generalization ability [23].

However, clustering algorithm selection and parameter settings significantly impact the results. Poor clustering may not represent the true distribution of the majority-class samples, affecting classification accuracy.

### 3.3. Hybrid Sampling Strategy

Hybrid Sampling Strategy combines oversampling and undersampling to balance advantages and disadvantages. The advantage is to balance data distribution while avoiding overfitting and information loss; the disadvantage is higher implementation complexity, requiring careful parameter tuning.

For example, SMOTE + K-means Undersampling increases minority samples via SMOTE, then prunes atypical majority samples with K-means to remove noise and redundancy. Instance-Level Undersampling + Bag-Level Oversampling for breast cancer multi-label classification, undersamples suspicious lesion regions (filtering false positives) and adaptively oversamples image bags with true positives—achieving 99.5% micro-average accuracy on the Inbreast database [21].

### 3.4. Data Augmentation

#### 3.4.1. Traditional Image Transformation Augmentation

Traditional image transformation augmentation techniques improve sample diversity via explicit mathematical operations on pixels or transform domain coefficients, including rotation, scaling, cropping, and flipping. The advantage is to simulate images from different angles/sizes, improving model adaptability. The disadvantage is that excessive transformation causes distortion and loss of medical information (e.g., blurred lesions from over-scaling). According to the different operation spaces, they can be classified into spatial-domain and transform-domain methods:

**Spatial-Domain Methods**: directly operate on pixel grayscale or neighborhood relationships of images, including point operations, neighborhood operations, and color space operations. Point operations use grayscale transformations (mainly gamma transformation and logarithmic transformation) and histogram transformations (mainly GHE and CLAHE) to adjust the distribution of pixel values and improve contrast and brightness. Neighborhood transformation is divided into linear filtering and non-linear filtering (e.g., Gaussian filtering, median filtering and bilateral filtering). Color space transformation converts RGB images to color spaces such as HIS/HSV for independent processing of brightness or saturation. Literature indicates that pseudo-color enhancement can be used to mark calcification points in breast X-rays in red to highlight tiny lesions, and color balance can be used to adjust channel ratios to eliminate color cast.

**Transform-Domain Methods**: convert images to the frequency domain or other transform domains to manipulate coefficients, mainly utilizing Fourier transform or wavelet transform. Fourier transform is particularly suitable for scenarios such as motion artifacts, high-contrast structures, and uneven illumination, as it achieves noise suppression, edge enhancement, and illumination compensation [24]. Wavelet transform decomposes images into low-frequency approximation layers and high-frequency detail layers (in horizontal/vertical/diagonal directions) through multi-resolution decomposition, enhancing edges and textures while suppressing noise. It is especially suitable for lung nodule detection preprocessing, tiny structure enhancement, and soft tissue texture optimization [25]. In addition to Fourier and wavelet transforms, other methods (such as DCT and Radon transform) have also been applied in clinical fields.

#### 3.4.2. Generative Adversarial Networks (GANs) Augmentation

GANs consist of a generator and a discriminator that compete in a two-player game, enabling the synthesis of highly realistic images to augment minority class samples [26,27,28]. The generator is responsible for generating new images similar to real images, while the discriminator judges whether the generated images are real. From a data construction perspective, the generative process of GANs can be viewed as a form of data-level or feature-level fusion. The generator effectively fuses the learned distribution of real medical images with random latent space vectors, synthesizing novel samples that retain authenticity while increasing diversity. This approach directly enriches the training set at a fundamental level. In liver tumor MRI image classification, GANs were used to generate more liver tumor image samples to supplement the number of minority-class samples [3]. For rare diseases, GANs have been used to generate synthetic patient records and achieve better prediction accuracy across different patient groups [29].

Compared with traditional image transformation augmentation, samples generated by GANs exhibit higher diversity and are more aligned with the real data distribution. However, training GANs requires substantial computational resources and complex parameter tuning, which increases the difficulty of their application.

Meanwhile, the GAN training process is unstable and prone to the problem of mode collapse. Specifically, in multimodal data distributions, the generator’s loss function becomes non-convex with respect to its parameters. This causes the generator to become trapped in local minima, only capturing a subset of the data modes. For instance, in medical image synthesis, the generator may only produce images of common lesion morphologies while ignoring the characteristics of rare lesions. To address mode collapse, efforts have primarily focused on the following three approaches:

First, improving loss functions and regularization techniques. Currently, the main methods include Wasserstein GAN (WGAN) and R3GAN. WGAN resolves the gradient vanishing issue by introducing the Wasserstein distance to replace the JS divergence, and also provides a quantifiable training criterion [30]. The latest improved WGAN algorithm further incorporates regularization terms and cosine similarity loss. Compared with the traditional WGAN, it achieves a 3.01% increase in precision, a 4.00% increase in AUC, and a 5.46% increase in accuracy. When compared with single-classification models, the F1-scores for minority classes (Analysis, Backdoor, Worms, and Shellcode) are improved by 8.36–13.20%, 11.48–13.28%, 54.16–58.63%, and 28.67–42.85%, respectively [31]. R3GAN, based on a regularized relative GAN loss function and combined with zero-centered gradient penalty, enables stable training on datasets such as FFHQ and ImageNet, with generated image quality surpassing that of diffusion models.

Second, dynamic conditional generation and self-supervised learning. The DynGAN framework dynamically partitions the training set and introduces a conditional generation model to progressively cover the multimodal distribution of real data [32]. Self-synergistic diffusion GAN combines diffusion models with GANs, achieving image synthesis by smoothing the data distribution through self-generated data.

Third, mode collapse detection and prevention. In practical applications, a combination of visualization (e.g., t-SNE dimensionality reduction), statistical analysis (e.g., entropy calculation), and evaluation metrics (e.g., FID) is commonly used to comprehensively detect mode collapse and conduct multi-dimensional assessments. In terms of architecture optimization, techniques such as attention mechanisms and spectral normalization are introduced to enhance the generator’s ability to model diverse data modes [33]. For example, StyleGAN2 reduces mode collapse while maintaining performance through simplified architecture design [34,35]. Recent advances in diffusion models have shown promise in generating high-fidelity medical images while mitigating mode collapse, offering a viable alternative to GANs in data augmentation for imbalanced datasets.

The clinical validity of GAN-generated data remains debated. Physicians must balance synthetic data accuracy with patient privacy concerns. By leveraging data anonymization technology, anonymous medical images can be generated based on GANs. These images allow classifiers to be trained without revealing patients’ identities, and the performance of such classifiers can be comparable to those trained on real data. Meanwhile, differential privacy and homomorphic encryption techniques can be applied. Specifically, within a federated learning framework, Laplace noise injection and gradient clipping techniques are used to ensure privacy security during collaborative cross-institutional data modeling.

### 3.5. Ensemble Methods

Ensemble methods improve classification performance by combining the predictions of multiple base learners [36], a process that can be viewed as decision-level fusion. This strategy is particularly valuable in imbalanced and complex data scenarios, as it can balance the contributions from different classes and enhance robustness. Following this principle, two representative ensemble techniques are widely used.

#### 3.5.1. EasyEnsemble

EasyEnsemble divides the majority-class samples into subsets. Each subset is combined with minority-class samples to form a new training set, on which multiple classifiers are trained separately. Finally, the results of these classifiers are integrated through methods such as voting or averaging. This approach can fully utilize the information of majority-class samples, avoid the over-reliance of a single classifier on majority-class samples, and improve the model’s ability to identify minority-class samples.

In tumor lncRNA prediction, EasyEnsemble + LightGBM improved the sensitivity index and the recognition accuracy of minority positive samples (tumor lncRNAs), successfully identifies more potential tumor lncRNAs, and provides an interpretable tool (SHAP analysis) to reveal the contributions of different omics features, thus offering new evidence for early tumor screening and target development [37]. For early detection of aortic dissection, its accuracy exceeds 80%, demonstrating high clinical application value [38].

However, training multiple classifiers increases computational cost and time consumption. Moreover, the ensemble effect depends on the diversity among classifiers; if the classifiers are too similar, the ensemble effect may be unsatisfactory.

#### 3.5.2. BalanceCascade

BalanceCascade adopts a cascaded structure and trains multiple classifiers on the samples misclassified by the previous stages, with a focus on the misjudged minority-class samples. As the cascade progresses, the model’s attention to minority-class samples gradually increases, and its classification ability is continuously enhanced. In the auxiliary diagnosis of cardiovascular diseases, compared with 89% sensitivity of the EasyEnsemble model, BalanceCascade achieves 92% sensitivity and also shows better performance in terms of average AUC and G-mean [38].However, BalanceCascade is relatively sensitive to noise. If there are mislabeled samples in the data, such errors may be amplified continuously during the cascading process, affecting the final classification result. Meanwhile, since multiple classifiers need to be trained sequentially, the training time is relatively long, making it unsuitable for application scenarios with high time requirements.

All data-centric strategies have inherent trade-offs in multi-sensor medical imaging clinical application: Oversampling methods (SMOTE, ADASYN) improve minority sample diversity but risk introducing false cross-modality features and fail to address sensor-specific scarcity. In contrast, undersampling methods reduce computational cost but may lose critical sensor-specific feature information from majority class samples. Traditional augmentation is simple and interpretable but cannot generate cross-modality consistent features. GAN-based augmentation (especially CM-GANs) generates high-fidelity, cross-modality compatible samples; however, it requires massive computational resources and faces strict regulatory validation of clinical plausibility. Hybrid sampling strategies (SMOTE + K-means undersampling) achieve the best balance between model performance and clinical practicability for moderate-to-severe imbalance (1:20~1:50), with an average F1-score 8.9~12.3% higher than single sampling methods in multi-sensor tasks.

## 4. Model-Centric Strategies

Data-level strategies form the first line of defense against class imbalance. The choice among oversampling, undersampling, augmentation, and hybrid approaches hinges on specific dataset characteristics and clinical constraints. Model-level strategies mainly adjust algorithms or loss functions to enable the model to better learn the features of minority-class samples and improve its classification ability [39]. The choice between data-centric and model-centric strategies should be guided by dataset characteristics, clinical task requirements, and resource constraints. Table 1 provides a consolidated comparison to aid researchers in method selection.

### 4.1. Loss Function Adjustment

#### 4.1.1. Weighted Loss Functions

Among various loss function optimization approaches, weighted loss functions are the most fundamental and widely used, with the two most frequently applied variants, e.g., Weighted Cross-Entropy (WCE) and Balanced Cross-Entropy (BCE). WCE is to introduce a weight factor for each class based on the standard cross-entropy framework. Specifically, the weight assigned to the minority class is set greater than 1, while the weight for the majority class is set less than 1. This design ensures that mispredictions of minority-class samples result in a more significant loss. WCE is generally suitable for all basic imbalanced scenarios; reduces the missed diagnosis rates for scenarios with severe data imbalance. BCE automatically calculates weights using the number of effective samples, thereby avoiding the subjectivity associated with manual parameter tuning. In scenarios where the sample distribution is dynamic, BCE can adjust weights automatically to tackle the imbalanced data issue.

#### 4.1.2. Focal Loss Functions

When dealing with hard-to-classify samples, Focal Loss is commonly employed. Its core principle involves integrating a modulation factor into the cross-entropy framework to suppress the loss of easy-to-classify samples while amplifying the loss of hard-to-classify samples. This approach is often used in visual medical tasks that demand high model discrimination capabilities, such as image-based diagnosis and pathological section classification.

#### 4.1.3. Probability Distribution Correction Loss Functions

Probability distribution correction loss functions are utilized to optimize the rationality of probability outputs from the model and enhance the clinical reliability of prediction results by correcting the probability distribution of the model’s outputs. A representative method of this type is the Weighted Mean Squared Error (WMSE) loss. WMSE assigns higher weights for high-risk samples (a subset of the minority class). This loss function is frequently applied in disease risk assessment and prognosis prediction, as it ensures smaller prediction errors for high-risk patients and prevents treatment delays caused by underestimated risks.

#### 4.1.4. Hybrid Loss Functions

In some more complex cases, strategies that combine multiple loss functions are also adopted. One notable method is the Unified Focal Loss, which generalizes Dice loss and cross-entropy-based loss to effectively manage class imbalance in medical image segmentation tasks. This loss function has been proven to outperform traditional loss functions because it is robust to class imbalance, thereby improving model convergence and segmentation accuracy [40]. Another innovative approach is the adaptive deep metric learning loss function, known as IDID-loss, which addresses the dual challenges of data scarcity and data density in class-imbalanced learning. This loss function generates diverse features within classes while preserving the semantic correlation between different classes and effectively reduces data overlap and density issues, thereby improving classification performance across various datasets [41]. Furthermore, asymmetric variants of popular loss functions (such as large-margin loss) have been proposed to counteract the logit shift phenomenon observed in neural networks for image segmentation tasks. These modifications aim to enhance the model’s generalization ability, particularly for underrepresented classes, thereby improving overall segmentation accuracy [42].

Although adjusting loss functions can solve the imbalanced data problem to a certain extent, it still has the following shortcomings: (1) The setting of hyperparameters/weights relies on experience, which easily introduces subjective bias; (2) It may exacerbate the instability of hard-to-classify sample learning; (3) It has poor adaptability in multi-task or multi-type scenarios; (4) There is a risk of poor model calibration; (5) It increases model complexity or parameter tuning costs. In the future, loss function models will develop toward dynamic adaptive loss, multi-task and multi-modal fusion, improved interpretability and robustness, and the realization of neural architecture search.

### 4.2. Transfer Learning

Transfer learning applies features learned from large-scale, balanced datasets to target datasets, and is particularly suitable for small and imbalanced datasets. In medical image classification, pre-trained deep learning models can capture the general features of images, which are then fine-tuned to adapt to the classification task of minority classes. In the healthcare field, transfer learning can enhance the classification of rare diseases by using models pre-trained on larger and more balanced datasets, which enables the extraction of relevant features, which can be fine-tuned on imbalanced datasets to improve the model’s ability to identify minority classes, as seen in chronic disease prediction models [29]. This not only improves the model’s accuracy but also ensures the model is robust to noise and variability in real-world data [43].

For multi-sensor medical imaging (e.g., PET-CT, MRI-ultrasound fusion), transfer learning achieves feature fusion at the pre-training stage: pre-trained models are trained on large-scale multi-sensor fusion image datasets (e.g., the TCIA multi-modality cancer dataset) to learn cross-modality shared features, then fine-tuned on the target imbalanced dataset with modality-specific feature extractors. This strategy effectively solves the problem of insufficient minority samples in single-sensor data, and the model accuracy is improved by 10.2–14.5% compared with transfer learning based on single-modality pre-training [44].

However, transfer learning also has inherent shortcomings and limitations. First, negative transfer may occur if the source and target domains differ significantly, degrading target task performance. The latest solution to this issue is the application of a general filtering framework, which dynamically suppresses interference from irrelevant source data through adversarial networks. Second, the effectiveness of transfer learning highly depends on the similarity between the source domain and the target domain (e.g., data distribution, feature space). Excessive differences can lead to the benefits being less than the costs, which is believed to be directly related to the transfer distance (a measure of distribution difference) and the target generalization error. A typical strategy to address this problem is the application of Domain-Adversarial Neural Networks (DANN). Third, transfer learning has high requirements for the quality of source data; biases, noise, or scenario deviations in the source data can directly contaminate the target model. Furthermore, transfer learning may cause overfitting and catastrophic forgetting when the target data is insufficient. Additionally, the “black-box” nature of transfer learning itself results in poor interpretability.

### 4.3. Ensemble Learning

Ensemble learning trains multiple classifiers and combines their prediction results to improve overall classification performance. Especially in imbalanced data scenarios, ensemble learning can effectively reduce the bias toward the majority class through the synergy of different classifiers. For example, Boosting and Bagging methods generate multiple weak classifiers and combine their predictions to improve the classification performance of minority classes.

Recent studies have demonstrated the effectiveness of ensemble learning in addressing class imbalance. For instance, a method that combines pre-trained models with transfer learning has shown promising results in breast cancer classification, achieving high accuracy despite inherent class imbalance in the dataset [45]. A hybrid model that integrates expert knowledge and advanced sampling techniques has been proposed to enhance the classification of uremic patients, effectively addressing the challenges posed by imbalanced data distribution [46]. The application of deep reinforcement learning, can adaptively focus on minority classes during training, thereby achieving better classification results in complex datasets.

Nevertheless, ensemble learning still has some shortcomings, such as: (1) high computational cost and resource consumption; (2) poor model interpretability; (3) sensitivity to noise and outliers, with a risk of overfitting; (4) complex hyperparameter tuning, leading to high implementation difficulty; (5) strong dependence on the quality of base classifiers—“weak ensembles” may fail, and it is not suitable for edge devices.

### 4.4. Multi-Task Learning

Multi-Task Learning (MTL) trains multiple related tasks simultaneously, sharing feature extraction layers to improve the model classification ability. Recent advances indicate that leveraging information from multiple related tasks holds promise for improving classification performance on imbalanced datasets. For example, MTKSVCR model that utilizes a multi-task multi-class support vector machine framework and incorporates a one-vs-rest strategy allows for better utilization of sample information and addresses class imbalance through customized regularization techniques. The model not only improves test accuracy but also introduces safe acceleration rules to enhance computational efficiency, making it suitable for large-scale applications [47]. CluSMOTE, which combines clustering and oversampling techniques, has improved the prediction of conformational B-cell epitopes by effectively balancing the dataset [48].

The shortcomings of MTL mainly focus on its strong dependence on task relevance and high risk of negative transfer. Furthermore, due to the inherent characteristics of its algorithm, the challenge of balancing parameter sharing and task specificity remains to be resolved. Essentially, the limitations of MTL stem from the complex coupling of three factors: “task relevance,” “resource allocation,” and “optimization synergy.” In scenarios where tasks are independent, data is extremely imbalanced, or high interpretability is required, MTL may be less effective than single-task learning or other strategies.

### 4.5. Model Calibration

Model calibration is to adjust the confidence of model predictions. Especially in imbalanced data scenarios, uncalibrated models may produce overconfident predictions that are biased toward the majority class. Through calibration, the model can generate more realistic and reliable prediction confidence, reducing the risk of misclassification. Model calibration algorithms have been applied to transition medical AI from research to clinical practice. For example, A. Carinelli systematically compared the performance differences in mainstream calibration methods (e.g., Logistic calibration, Beta calibration, regression transformation-based calibration) in transferred populations through simulation experiments [49]. The results indicated that regression methods based on transformed probability estimation (e.g., probability calibration after logit transformation) outperform raw probability calibration, especially in scenarios with changing data distributions (e.g., cross-center medical data), providing theoretical support for model transfer calibration in multi-center clinical trials or medical scenarios across different regions. A Chinese multi-center research team developed a risk prediction model for distant metastasis of papillary thyroid cancer (PTC) using 9 algorithms (including random forest and XGBoost). The reliability of the model was evaluated through calibration curves, Brier scores, and the Hosmer-Lemeshow test, enabling its clinical application and supporting clinicians in dynamically adjusting threshold-based decision-making for intervention intensity [50]. In the task of cardiac CMR artifact detection, studies have shown that combining ENN with UvAC Loss can make the calibration curve approach the diagonal line [51]. Calibration is particularly critical in multi-sensor imaging, where overconfident predictions from an uncalibrated model may lead clinicians to disregard potentially correct minority-class predictions from complementary modalities.

However, model calibration has limitations. Calibration and accuracy may conflict, and calibration performance degrades under data distribution shifts or small sample sizes. Moreover, calibration methods themselves have inherent flaws, and evaluation metrics have limitations. For instance, Expected Calibration Error (ECE) estimates calibration error through binning, and the binning method (e.g., equal width, equal frequency) affects the stability of results and makes it difficult to capture fine-grained calibration biases; the Brier score includes both calibration error and discrimination error, so it cannot reflect calibration quality independently and may mask the true calibration problems of the model [52]. For multi-sensor imaging, calibration must be evaluated both per-modality and post-fusion. Metrics such as Modality-Specific Expected Calibration Error (MS-ECE) and Fusion Calibration Error (FCE) can be introduced to assess calibration consistency across sensors and after fusion. Cross-modality calibration consistency, measured by the variance of per-modality ECE, is also critical for reliable multi-modal diagnosis.

**Table 1:** Comprehensive Comparison of Key Methods for Imbalanced Medical Image Classification.

Category	Specific Method	Core Mechanism	Advantages	Disadvantages/ Clinical Risks	Typical Medical Imaging Application	Optimal Imbalance Ratio
Over- sampling	SMOTE	Generates synthetic minority samples by interpolating between existing ones in feature space.	Increases diversity of minority class, mitigates overfitting from simple duplication.	May create unrealistic samples; effectiveness can diminish with high-dimensional image features.	Benign/Malignant thyroid nodule classification [8].	1:4~1:20
	Borderline- SMOTE	Oversamples only the minority samples near the decision boundary.	Strengthens the decision boundary, improves model discrimination for borderline cases.	Sensitive to boundary definition; higher computational complexity.	Tasks requiring fine-grained boundary discrimination (e.g., early-stage lesion identification) [10].	1:10~1:30
	ADASYN	Adaptively generates synthetic samples based on learning difficulty, focusing on harder samples.	Targets hard-to-classify regions, potentially boosting model performance where needed.	Inaccurate difficulty estimation can introduce noisy samples.	Early Alzheimer’s disease diagnosis; classification of small breast cancer lesions [9,20].	1:20~1:50
Undersampling	Cluster-based (e.g., K-means)	Clusters majority class samples first, then selects representative samples from each cluster.	Preserves the distribution structure of the majority class, minimizing information loss.	Results heavily depend on clustering algorithm and parameter choices.	Diabetes prediction [53]	1:4~1:30
Data Augmentation	Traditional (Rotation, Flip, etc.)	Applies spatial/transform-domain transformations to images.	Simple, efficient, interpretable; simulates different acquisition conditions.	May cause distortion or loss of critical diagnostic features if over-applied.	General-purpose augmentation for X-ray, CT image classification [54]	1:4~1:20 (mild imbalance)
	Generative Adversarial Networks (GANs)	Uses a generator to synthesize realistic minority-class medical images.	High sample diversity and fidelity, closely mimics real data distribution.	Training instability (mode collapse), high computational cost, clinical validity of generated samples needs verification.	Liver tumor MRI data expansion; rare disease image synthesis [3].	>1:50 (extreme)
Ensemble Learning	Easy- Ensemble	Divides majority class into subsets, each combined with all minority samples to train multiple classifiers, then ensembles results.	Makes full use of majority class information; improves stability and minority class recall.	High computational cost and time consumption from training multiple classifiers.	Tumor biomarker prediction; early detection of aortic dissection [37,38].	1:10~1:40
	Balance- Cascade	Uses a cascade structure; each classifier is trained on samples misclassified by the previous one, focusing on remaining hard samples.	Progressively focuses on difficult samples, achieving high sensitivity for the minority class.	Sensitive to label noise; errors can amplify through cascade; long training time.	Cardiovascular disease diagnosis (where high sensitivity is critical) [38].	1:10~1:50
Loss Function	Weighted Cross- Entropy (WCE/BCE)	Assigns higher loss weights to the minority class during training.	Direct and effective, forces the model to pay more attention to the minority class.	Weight setting is empirical and subjective; may degrade model calibration.	Foundational adjustment strategy for various medical image classification tasks [41].	1:4~1:50
	Focal Loss	Reduces the loss contribution from easy-to-classify samples, focusing the model on hard ones.	Particularly effective for tasks with abundant simple negatives (e.g., background in detection).	Introduces additional hyperparameters, complicating optimization.	Lesion detection and segmentation in medical images [40].	1:10~1:50
Transfer Learning	Pre-trained Model Fine-tuning	Leverages features from models pre-trained on large-scale (natural/medical) image datasets, fine-tuned for the imbalanced target task.	Alleviates overfitting on small datasets; utilizes powerful generic feature extractors.	Risk of negative transfer if source/target domains differ significantly; poor interpretability.	Rare disease classification; model adaptation across centers and devices [43].	1:10~1:50

## 5. Clinical Deployment and Compliance Framework

The successful translation of imbalanced learning research into clinical practice hinges not only on algorithmic performance but also on seamless integration into medical workflows and adherence to stringent regulatory standards. This section outlines the holistic pathway from development to deployment, focusing on the operational workflow, key regulatory requirements, and persistent practical challenges.

### 5.1. Clinical Deployment Workflow

**Figure 3:**
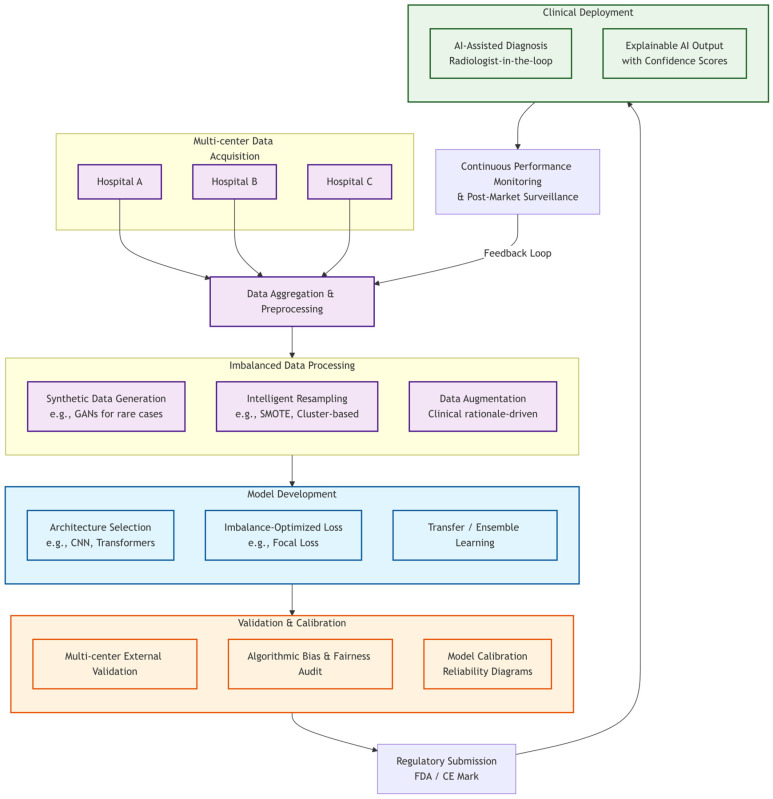
Clinical workflow integrating imbalance handling strategies for medical AI diagnosis. The vertically structured workflow begins with multi-center data acquisition, followed by imbalance-aware data processing, model development with specialized architectures and loss functions, rigorous validation and calibration, regulatory submission, clinical deployment with radiologist-in-the-loop, and continuous monitoring forming a feedback loop for ongoing improvement.

The rapid development of medical AI technology brings great potential to analyze and process medical data to assist in medical decision-making, disease diagnosis, treatment plan formulation and other medical activities. Clinically, AI-assisted diagnostic systems accelerate medical image screening, while population health data analysis enables disease risk prediction and personalized health advice. A clinically viable AI diagnostic tool must navigate a multi-stage pipeline that explicitly addresses data imbalance at critical junctures. As illustrated in Figure 3, this workflow integrates technical solutions with clinical and regulatory checkpoints:

(1) Multi-Center Data Acquisition and Imbalance-Aware Processing: The pipeline begins with aggregating imaging data from diverse sources (Hospitals A, B, C). This inherently imbalanced dataset then undergoes specialized preprocessing, including synthetic data generation (e.g., GANs for rare cases), intelligent resampling (e.g., SMOTE), and clinically rational data augmentation to create a more balanced and robust training set.
(2) Model Development and Validation: Subsequently, models are trained using imbalance-optimized architectures and loss functions (e.g., Focal Loss, transfer learning). Crucially, validation must be multi-centered and include bias/fairness audits and model calibration to ensure reliability and generalizability beyond the development dataset.
(3) Regulatory Submission and Clinical Integration: Following rigorous validation, the model undergoes regulatory review (e.g., FDA, CE Mark). Upon approval, it is deployed in a radiologist-in-the-loop setting, providing explainable outputs with confidence scores to support clinical decision-making.
(4) Continuous Monitoring: Post-deployment, continuous performance monitoring and post-market surveillance create a feedback loop, enabling ongoing refinement and ensuring sustained safety and efficacy in real-world use.

### 5.2. Regulatory Compliance for Medical AI

Due to the special nature of the medical field, involving the life and health of patients, the application of medical AI must be carried out under strict regulatory frameworks to ensure its safety, efficacy, and reliability (Table 2). As applications expand, compliance challenges emerge. Regulators worldwide have strengthened supervision, with the U.S. Food and Drug Administration (FDA) system as the mainstream certification framework. Understanding and following these requirements is key to the successful entry of medical AI products into the market and their widespread adoption. For clinical deployment and global market access, compliance with regulatory frameworks such as the FDA is paramount.

#### 5.2.1. Standard Requirements for Medical Imaging Datasets

For medical imaging datasets used in imbalanced learning and clinical AI deployment, the FDA and EU MDR have established unified quantitative and qualitative standards, which are core to regulatory submission:

(1) Sample diversity standard: The dataset must include samples from at least 3 multi-center clinical sites, covering different age groups (18–80+ years), genders, and ethnicities, with the proportion of minority groups (e.g., ethnic minorities, pediatric/geriatric patients) not less than 15% to avoid demographic bias.
(2) Imaging quality standard: All medical images must meet the clinical diagnostic quality requirements of the corresponding modality (e.g., CT spatial resolution ≥0.625 mm, MRI signal-to-noise ratio ≥20), with a quality control pass rate of 100% verified by two senior radiologists.
(3) Annotation standard: Lesion annotation must be completed by at least two board-certified radiologists with inter-annotator agreement (Cohen’s kappa) ≥0.85; ambiguous annotations must be resolved through a third expert review.
(4) Data management standard: Datasets must include complete metadata (imaging protocol, patient clinical information, annotation time), and raw data must be stored in DICOM 3.0 standard format with traceable version control.
(5) Synthetic data standard: For synthetic data generated by GANs/SMOTE, the similarity with real clinical data (assessed by Fréchet Inception Distance (FID) for 2D images, Dice Similarity Coefficient (DSC) for 3D images) must be ≥0.90, and clinical plausibility must be verified by a clinical expert panel.

#### 5.2.2. FDA Certification Processes

The FDA uses a hierarchical regulatory model based on risk and functionality and classifies medical AI products based on the risk level of medical device functionality and the importance of the use case. Following FDA certification processes, possible product issues could be detected and dealt with in a timely manner:

(1) 510(k) programs: For low- to medium-risk medical AI products substantially equivalent to marketed “predicate devices”. Manufacturers are required to conduct sufficient product testing and verification, including performance testing, safety testing, etc., before submitting applications. The application materials should include a product description, intended use, comparative analysis with the “predicate device”, test report, etc. FDA usually completes the review within 3–6 months.
(2) De Novo Procedure: For novel low- and medium-risk devices incompatible with existing market medical devices. Manufacturers need to provide proof of safety and efficacy, as well as establish classification rules. FDA review time is typically 150 business days.
(3) PMA Program: For high-risk medical AI products. Manufacturers need to conduct large-scale, multi-center clinical trials to collect sufficient clinical data to demonstrate the safety and efficacy of the products. Application materials include detailed product design, manufacturing process, clinical research report, risk management plan, etc. FDA review time is typically 180 business days.

Throughout these processes, the FDA emphasizes explainability, demands training data that covers diverse populations to mitigate bias, and requires plans for post-market performance monitoring. The choice of imbalance-handling strategy directly impacts the regulatory submission. The integration of synthetic data into clinical workflows necessitates rigorous validation against real-world multi-center datasets to ensure generalizability and compliance with FDA guidelines on algorithmic fairness and data provenance. Table 2 summarizes key FDA considerations for different method categories. For instance, using synthetic data (GANs/SMOTE) necessitates demonstrating clinical plausibility and absence of deceptive artifacts, while ensemble methods require stability analysis and real-time performance validation.

### 5.3. Critical Challenges and Solutions

Despite comprehensive strategies, which combine multiple technologies at the data and model levels, have greatly improved classification performance in imbalanced data scenarios in certain specific tasks, they still face challenges in broader practical applications. To more effectively address the problem of imbalanced classification in medical images, the field must evolve from developing isolated algorithms to engineering integrated, robust, and clinically trustworthy systems. We envision several convergent pathways:

(1) Federated Learning and Privacy-Preserving Synthesis: Models trained on data from one hospital network may perform poorly on data from another due to differences in imaging equipment, protocols, and patient demographics—a challenge magnified when using synthetic or augmented data. The combination of Federated Learning (FL) and synthetic data generation (e.g., GANs) presents a paradigm shift [55,56]. FL can leverage distributed data across institutions without sharing raw images, while on-device or server-assisted GANs can generate site-specific synthetic minority samples. Future research must address challenges like cross-site distribution shifts in generated data and the development of efficient algorithms (like FedSPU [55]) for personalized and robust model training in resource-constrained environments.
(2) Explainable AI for Imbalanced Learning: As models become more complex, ensuring their decisions are interpretable and clinically plausible is non-negotiable. Future methods should tightly integrate Explainable AI (XAI) techniques like SHAP or attention maps directly into the training loop of imbalanced classifiers. This will allow clinicians to verify whether the model’s increased sensitivity to a minority class stems from medically relevant features or spurious correlations in synthesized/augmented data.
(3) Foundation Models and Few-Shot Learning Adaptation: The emergence of large-scale, pre-trained foundation models for medical imaging offers a promising alternative to traditional transfer learning. There is often a tension between methods that achieve high accuracy (e.g., complex GANs, deep ensembles) and those that meet clinical needs for speed, interpretability, and regulatory simplicity. By leveraging their rich, general-purpose visual representations, adaptation to new, imbalanced tasks could be achieved through efficient fine-tuning or prompt-based learning with very few examples, potentially bypassing the need for extensive data augmentation or resampling [57].

In multi-sensor medical imaging, the integration of imbalance-aware learning with fusion frameworks faces unique clinical deployment challenges:

(1) Cross-modality feature misalignment: Imbalance-handling methods for a single modality may disrupt feature consistency across sensors, leading to fusion performance degradation;
(2) Real-time fusion computational cost: Complex imbalance-handling methods (e.g., CM-GANs, cascaded ensembles) increase model complexity, making real-time fusion difficult on clinical hardware (e.g., radiology workstations);
(3) Regulatory validation of fusion models: The FDA/MDR require separate validation of each sensor modality and the integrated fusion model, with imbalance-handling effects verified for both single-modality and fusion results;
(4) Interpretability of fusion-imbalance models: Cross-modality feature fusion and imbalance-handling together exacerbate the “black-box” problem, making it difficult to trace the model’s minority class prediction to specific sensor features.
(5) To address these challenges, future research must develop sensor-adaptive imbalance-handling methods with low computational cost, and integrate XAI techniques (e.g., cross-modality attention maps) to improve interpretability.

## 6. Conclusions

Effectively addressing class imbalance is critical for developing reliable and equitable AI in medical imaging. This review has systematically categorized solutions into two paradigms: data-centric strategies that manipulate training distributions (e.g., resampling, augmentation, GANs), and model-centric strategies that enhance algorithmic resilience (e.g., tailored loss functions, transfer learning, ensembles).

We note a persistent tension: high-performance methods (e.g., complex GANs, cascaded ensembles) often face clinical deployment challenges due to complexity, interpretability gaps, and regulatory barriers. Therefore, the choice of strategy must be context-driven, balancing the clinical task, data landscape, and compliance requirements. Future research should prioritize integrating explainable AI with imbalance-aware learning—ensuring that the model’s focus on minority classes is based on clinically relevant features and meets FDA’s explainability requirements for medical AI. We also advocate for developing federated learning frameworks that preserve privacy while handling imbalance, which enable cross-institutional multi-center data collaboration without sharing raw medical images, and are fully compliant with HIPAA and EU GDPR data privacy regulations. Additionally, leveraging foundation models for few-shot adaptation in multi-sensor medical imaging can bypass extensive data augmentation by learning cross-modality universal features, which is critical for real-world clinical deployment in resource-constrained medical institutions. Notably, multi-sensor fusion currently faces open challenges including cross-modality feature misalignment, high computational cost of real-time fusion, and inconsistent calibration across different imaging sensors, which require further algorithmic optimization and clinical validation.

## Figures and Tables

**Table 2:** Key Compliance Considerations for FDA Certification of Imbalanced Learning Methods in Medical AI.

Method Category	Key FDA Certification Considerations and Validation Requirements
All Methods	1. Performance Validation: Provide performance reports (Sensitivity, Specificity, AUC, etc.) on multi-center, prospective clinical datasets. 2. Bias Assessment: Must evaluate and demonstrate algorithmic fairness across demographic subgroups (age, gender, ethnicity). 3. Traceability: Complete logs of model development, data preprocessing, and augmentation steps.
Synthetic Data Generation (e.g., SMOTE, GANs)	1. Realism Verification: Must demonstrate clinical plausibility of synthetic samples, typically requiring expert review or Turing-test-like evaluation. 2. Generalizability Testing: The model trained with synthetic data must be tested on an independent, entirely real clinical dataset to prove performance gains are not artifacts of synthesis. 3. Privacy Compliance: If real data is used for generation, ensure compliance with regulations like HIPAA, providing proof of anonymization.
Data Augmentation	1. Transformation Justification: All image transformations must have clear clinical rationale (e.g., simulating different view angles) with safe intensity thresholds to prevent distortion of diagnostic features. 2. Consistency Testing: Diagnostic decisions from models trained on augmented data should show high agreement with those from models trained on original data for critical cases.
Ensemble/Cascade Models	1. Stability Report: Provide performance and stability analysis for each base classifier and the ensemble, demonstrating robustness of the strategy. 2. Real-time Performance Validation: For cascade models, inference time must be tested on target clinical hardware to ensure it meets real-time diagnostic requirements. 3. Error Propagation Analysis: For methods like BalanceCascade, analyze and mitigate the risk of error amplification through the cascade stages.
Model Calibration	1. Calibration Curves: Must provide calibration curves (reliability diagrams) on validation and test sets, and compute metrics like Expected Calibration Error (ECE). 2. Clinical Decision Support: Calibrated probability outputs should align with clinical risk stratification, providing reliable decision thresholds for physicians.

## Data Availability

No new data were created or analyzed in this study.

## Abbreviations

The following abbreviations are used in this manuscript:

SMOTE	Synthetic Minority Over-sampling Technique
ADASYN	Adaptive Synthetic Sampling Approach
GANs	Generative Adversarial Networks
FDA	U.S. Food and Drug Administration
WCE	Weighted Cross-Entropy
BCE	Balanced Cross-Entropy
FL	Focal Loss
MTL	Multi-Task Learning
DANN	Domain-Adversarial Neural Networks
ECE	Expected Calibration Error
PTC	Papillary Thyroid Cancer
FedSPU	Federated Learning with Stochastic Parameter Update
ERI	Effort-Reward Imbalance
